# Effects of stocking density on growth, physiological, and puberty responses of replacement beef heifers reared in drylots

**DOI:** 10.1093/jas/skag151

**Published:** 2026-05-09

**Authors:** Camila P Prado, Reinaldo F Cooke, Shea J Mackey, Autumn T Pickett, Guilherme A Monteiro, Izadora S De Souza, Courtney L Daigle, Kelsey M Harvey

**Affiliations:** Department of Animal Science, Texas A&M University, College Station, TX 77843, United States; Department of Animal Science, Texas A&M University, College Station, TX 77843, United States; Department of Animal Science, Texas A&M University, College Station, TX 77843, United States; Department of Animal Science, Texas A&M University, College Station, TX 77843, United States; Department of Animal Science, Texas A&M University, College Station, TX 77843, United States; Department of Animal Science, Texas A&M University, College Station, TX 77843, United States; Department of Animal Science, Texas A&M University, College Station, TX 77843, United States; Prairie Research Unit, Mississippi State University, Prairie, MS 39756, United States

**Keywords:** beef heifers, puberty, stocking density, stress

## Abstract

This experiment evaluated growth, physiological responses, and puberty attainment in heifers reared in drylots with different stocking densities. A total of 240 heifers (75% Angus × 25% Brahman) were used. Heifers were ranked by age (270 ± 1 d), body weight (BW on day −3; 232 ± 2 kg) and temperament score on day 0, and assigned to drylot pens with: 1) 14 m^2^/heifer (HDENS), 2) 28 m^2^/heifer (MDENS), 3) 42 m^2^/heifer (LDENS), or to pastures with 1380 m^2^/heifer (CON). Each pen or pasture (*n* = 6/treatment) housed 10 heifers. Negligible forage was available for CON, and all treatments received the same limit-fed diet (∼9 kg/heifer daily, dry matter basis). Shrunk BW was recorded on day −3 and 171 to calculate BW gain. Heifers were fitted with an ear tag on day 0 to record behavioral responses. Blood samples were collected weekly for plasma progesterone analysis. Whole blood samples were collected on day 0, 58, 114, and 170 for mRNA isolation. Hair samples from the tail switch were collected on day 0, 30, 58, 85, 114, 142, and 170. Data were analyzed with pen or pasture as experimental unit. No treatment effects were detected (*P *= 0.92) for BW gain (∼0.647 kg/d). Heifers from CON spent more time eating (*P* < 0.01) and less time in other activity categories (active, highly active, and non-active; *P *≤ 0.05) compared with heifers reared in drylots. Hair cortisol concentrations were greater (*P *≤ 0.02) for HDENS compared with CON and MDENS on day 30, greater (*P *≤ 0.04) for HDENS compared with all other treatments on days 58 and 142, and less (*P *≤ 0.05) for CON compared with all drylot treatments on day 170 (treatment × day; *P *= 0.08). Expression of *heat shock protein* (HSP)-70 mRNA was greater (*P *≤ 0.02) for HDENS compared with all other treatments on day 170 (treatment × day; *P *= 0.04). Expression of HSP-72 mRNA was greater (*P *< 0.01) for LDENS compared with all other treatments on day 114, and greater (*P *< 0.01) in HDENS compared with all other treatments on day 170 (treatment × day; *P *< 0.01). A greater (*P *≤ 0.05) proportion of CON were pubertal by week 22 of the experiment compared with all other treatments and compared with MDENS and HDENS on week 23 (treatment × day; *P *< 0.01). In summary, rearing heifers in drylots with high stocking density (14 m^2^/heifer) increased chronic stress and delayed puberty compared with pasture heifers, whereas reducing drylot stocking density (28 or 42 m^2^/heifer) attenuated stress responses but did not benefit heifer reproductive development.

## Teaser

Beef heifers reared in drylots with high stocking density experience increased chronic stress reactions, and delayed puberty compared with cohorts reared on pasture. Therefore, this experiment evaluated if decreasing the stocking density will alleviate stress responses and hasten reproductive development of beef heifers reared in drylots.

## Introduction

The U.S. beef industry faces the challenge of increasing productivity while promoting animal welfare, ecological stewardship, and the judicious use of natural resources (FAO 2009). Stocking density is a management decision that directly influences each of these objectives, but it is often overlooked in cow-calf systems due to their extensive production nature (Asem-Hiablie et al. 2017). Although increasing stocking density can enhance productivity with less land use (Lardy et al. 2017), such intensification is frequently accompanied by negative consequences to animal well-being (Fraser et al. 2013). Research from our group demonstrated that heifers reared in drylots with high stocking density (14 m^2^/heifer; HDENS) experienced chronic stress reactions elicited by confinement and restricted physical activity, resulting in delayed puberty attainment compared with cohorts managed on pastures with low stocking density (Schubach et al. 2017; Harvey et al. 2024). Acute and chronic stress responses impair reproductive development (Dobson and Smith 2000), whereas high stocking density is perceived as a major stressor by cattle (Huzzey et al. 2012). These findings supported the need for research-based recommendations regarding stocking density decisions for intensive heifer development programs (Tucker et al. 2015).

Despite being within the recommended values by FASS (2020), the HDENS treatment in Schubach et al. (2017) and Harvey et al. (2024) characterized a stocking density that was detrimental to well-being and development of replacement heifers. Across livestock species reared intensively, reducing stocking density typically improves behavioral responses, productivity, and welfare conditions (Estevez et al. 2007). Based on this rationale, we hypothesized that reducing stocking density would improve welfare, as reflected by physiological and behavioral indicators of stress, and enhance reproductive development of beef heifers reared in drylots. To test this hypothesis, this experiment compared behavioral, physiological, and reproductive responses in beef heifers reared on four different stocking densities; a negative control (14 m^2^/heifer, Schubach et al. 2017; Harvey et al. 2024), two experimental stocking densities (28 and 42 m^2^/heifer), and heifers reared on pasture (1,380 m^2^/heifer) as a positive control (Schubach et al. 2017; Harvey et al. 2024).

## Materials and methods

This experiment was conducted over 2 consecutive years (year 1: November 2023 to April 2024; year 2: November 2024 to April 2025) at the Texas A&M—McGregor Research Center (McGregor, TX). Heifers were cared for in accordance with acceptable practices and experimental protocols reviewed and approved by the Texas A&M AgriLife Research, Agriculture Animal Care and Use Committee (no. 2023-018A).

### Animals and treatments

A total of 240 heifers (75% Angus × 25% Brahman) born at the Texas A&M—McGregor Research Center (McGregor, TX) were used (120 heifers/year; day 0 to 170). Heifers were maintained on pasture with their respective dams from birth until weaning. Heifers were weaned 3 weeks prior to the beginning of the experiment and maintained in a single 10-ha pasture with ad libitum access to bermudagrass hay (*Cynodon dactylon*), water, and a commercial mineral and vitamin mix. On day 0, heifers were ranked by age (270 ± 1 d), shrunk body weight (BW on d −3; 232 ± 2 kg), and temperament score (Cooke 2014) on day 0 and assigned to 1) 1 of 6 drylot pens (10 × 14 m pens; 10 heifers/pen) with a stocking density of 14 m^2^/heifer (HDENS), 2) 1 of 6 drylot pens (10 × 28 m pens; 10 heifers/pen) with a stocking density of 28 m^2^/heifer (MDENS), 3) 1 of 6 drylot pens (10 × 42 m pens; 10 heifers/pen) with a stocking density of 42 m^2^/heifer (LDENS), or 4) 1 of 6 pastures (1.38-ha pastures; 10 heifers/pasture) with a stocking density of 1,380 m^2^/heifer (CON). All pastures were mowed prior to the beginning of the experiment and as necessary to ensure negligible forage was available for grazing to CON heifers, with residual forage clipping removed when necessary.

All heifers received the same limit-fed total mixed ration (TMR), which averaged 9 kg/heifer daily (dry matter basis) and had free-choice access to water during the experimental period (day 0 to 170). The TMR was offered at 0800 h daily in feed bunks with similar linear space across treatments (0.72 m/heifer) and were consumed within 6 h of feeding. The TMR included (dry matter basis) 44% of corn silage, 28% of sorghum-Sudan hay, 12% of ground corn, 9% of dried distillers’ grain, 5% of liquid molasses, and 2% of mineral mix. Nutritional profile of the TMR (dry matter basis) was 1.56 Mcal/kg of net energy for maintenance, 0.97 Mcal/kg of net energy for gain, and 13.2% of crude protein. The mineral mix contained 21% Ca, 0.01% P, 21% NaCl, 0.20% K, 0.10% Mg, 0.045% Cu, 0.001% Se, 0.280% Zn, 220,000 IU/kg of vitamin A, 19,800 IU/kg of vitamin D3, and 3,500 IU/kg of vitamin E (Anipro Xtraperformance Feeds, College Station, TX), as well as sodium monensin (Rumensin; Elanco Animal Health, Greenfield, IN) at 1,320 g/ton (37.2 g/ton of TMR, dry matter basis).

### Sampling

Heifer shrunk BW was recorded after 16 h of feed water withdrawal on days −3 and 171 to represent initial and final BW, respectively, and used to calculate average daily gain (ADG). Heifer temperament was assessed via chute score and exit velocity as described by Cooke (2014) on days 0, 86, and 170. Heifers were fitted with an ear tag (CowManager, Select Sires, Plain City, OH) on day 0 of the experiment to record behavioral responses, including time spent ruminating, eating, and being physically active or inactive (Wolfger et al. 2015; Pereira et al. 2018). Heifer full BW was recorded, and blood samples were collected weekly during the experiment (days 0 to 170). Blood was collected via jugular venipuncture into commercial blood collection tubes containing freeze-dried sodium heparin (Vacutainer, 6 mL; Becton Dickinson, Franklin Lakes, NJ) for plasma collection. Data from the CowManager system recorded during sampling days were discarded to eliminate the confounding effects of gathering, handling, and processing on physical activity. Growth rate of each heifer was modeled by linear regression of full BW against sampling days, and each regression coefficient was used as an individual growth response.

Heifers from all treatments were allowed to rest for 1 h in the working facility prior to processing and sample collection (Harvey et al. 2024). Additional blood samples were collected via jugular venipuncture into commercial blood collection tubes containing ethylenediamine triacetic acid (Vacutainer, 6 mL; Becton Dickinson) for whole blood mRNA isolation (Pohler et al. 2017) on d 0, 58, 114, and 170. Hair samples were collected from the tail switch of all heifers on d 0, 30, 58, 85, 114, 142, and 170 using scissors as close to the skin as possible (Schubach et al. 2017). Within each sampling, hair was collected from an area that had not been previously sampled, and hair material closest to the skin was stored (1 cm of length and ∼100 mg of weight).

### Laboratorial analyses

#### Blood samples

All blood samples were immediately placed on ice after collection, centrifuged (2.500 × g for 30 min; 4 °C) for plasma or white blood cell harvest, and stored at −80 °C on the same day of collection. Plasma samples collected weekly were analyzed for progesterone concentrations (radioimmunoassay kit #07-170105, MP Biomedicals, Santa Ana, CA; Pohler et al. 2016). Heifers were considered pubertal once plasma progesterone concentrations were ≥ 1.5 ng/mL followed by a cyclic pattern of plasma progesterone and < and ≥ 1.5 ng/mL suggestive of normal estrous cycles (Cooke and Arthington 2009). Heifer age and BW at puberty were calculated based on weekly full BW measurements and heifer age at the week of puberty attainment. The intra- and inter-assay CV were 6.09% and 3.44%, respectively.

For blood samples collected for mRNA isolation, plasma was decanted and white blood cells were collected by buffy coat extraction (Pohler et al. 2017), placed in a 2 mL cryotube with 1 mL of TRIzol solution (Invitrogen, Carlsbad, CA), and stored at −80°C on the same day of collection. Total RNA was extracted using the TRIzol Plus RNA purification kit (Invitrogen). Quantity and quality of isolated RNA were assessed via UV absorbance (NanoDrop Lite; ThermoFisher Scientific, Wilmington, DE) and 260 nm and 260/280 nm ratio, respectively. Reverse transcription of extracted RNA and real-time reverse-transcription polymerase chain reaction (PCR) were performed using gene-specific primers (500 nM/reaction of each primer; Table 1) and the Fast SYBR Green PCR Master Mix (Thermo Fisher Scientific) with the QuantStudio 3 Real-Time PCR System (Thermo Fisher Scientific), as previously described by Rodrigues et al. (2015). Responses from the genes of interest were quantified based on the threshold cycle (CT), the number of PCR cycles required for target amplification to reach a predetermined threshold. A portion of the amplified product was purified with the QIAquick PCR Purification Kit (Qiagen Inc., Valencia, CA) and sequenced at the Texas A&M—College of Veterinary Medicine and Biomedical Sciences to verify amplification specificity and confirm that the expected target transcripts were amplified. All amplified products represented only genes of interest. Amplification performance was consistent across samples, supporting the use of these primers for relative quantification under the conditions used herein, consistent with approaches used in similar studies (Schubach et al. 2017; Harvey et al. 2024). The CT responses from genes of interest were normalized by the geometrical mean of CT values of *ribosomal protein 9* and *β-actin* (Vandesompele et al. 2002). The CV for the geometrical means of reference genes across all samples was 4.10%. Results are expressed as relative fold change (2^ΔΔ^CT; Ocón-Grove et al. 2008).

**Table 1:** Primer sequences, accession number and reference for all gene transcripts analyzed by real-time reverse transcription PCR.

Target^1^	Primer sequence	Accession no. and reference
**HSP-70**		
**Forward**	CGGCTTAGTCCGTGAGAACA	BTU09861
**Reverse**	CCGCTCGGTATCGGTGAA	Liu et al. (2014)
**HSP-72**		
**Forward**	AACATGAAGAGCGCCGTGGAGG	U02892
**Reverse**	GTTACACACCTGCTCCAGCTC	Lacetera et al. (2006)
**B2M**		
**Forward**	ACATCCCGTCCTTCATCGT	NM_173893
**Reverse**	GCCCTTCTTGGCGTTCTT	Rodrigues et al. (2015)
**RSP9**		
**Forward**	GGGCTGCTGTCGCTGTCT	NM001101152
**Reverse**	TCTTCTGGTGGGTGTCTTGAGT	Liu et al. (2014)

1 HSP-70 = *heat shock protein 70*; HSP-72 = *heat shock protein 72*; B2M = *ß-microglobulin*; RSP9 = *ribosomal protein S9.*

#### Hair samples

Cortisol was extracted from hair samples as in Schubach et al. (2017). Samples were reconstituted in 100 µL of phosphate-buffered saline supplied with an enzyme-linked immunoassay cortisol kit (Salimetrics Expanded Range, High Sensitivity 1-E3002, State College, PA) and stored at −80 °C. Samples were analyzed for cortisol concentrations using the aforementioned enzyme-linked immunoassay kit. The intra- and inter-assay CV were 5.12 and 5.77%, respectively.

### Statistical analysis

All data were analyzed using drylot pen or pasture (6 replications/treatment) as an experimental unit, and replication (treatment × year), heifer (replication), and year as random variables. Quantitative data were analyzed using the MIXED procedure of SAS (SAS Inst. Inc., Cary, NC), whereas binary data were analyzed using the GLIMMIX procedure of SAS. All data were analyzed using the Satterthwaite approximation to determine the denominator degrees of freedom for tests of fixed effects. Model statements used for heifer ADG, initial and final BW, growth rate, temperament variables, and BW and age at puberty contained the effects of treatment. Model statements for puberty attainment, physical activity, and physiological variables contained the effects of treatment, day, and the treatment × day interaction. Blood and hair variables were analyzed using the result from d 0 as an independent covariate. The specified term for the repeated statement was day and the subject was heifer (replication). Covariance structures tested included variance components, compound symmetry, unstructured, and autoregressive, with the autoregressive structure selected based on the lowest Akaike information criterion. All results are reported as least square means, or covariately adjusted least square means for blood and hair variables, and separated using PDIFF adjusted to the Tukey–Kramer method to prevent Type I errors. Significance for differences were set at *P *≤ 0.05 and tendencies were determined if *P *> 0.05 and ≤ 0.10. Results are reported according to the main effect of treatment when the treatment × day interaction was not significant (*P *> 0.10).

## Results and discussion

Despite the relevance of stocking density for livestock welfare and productive efficiency, limited research has evaluated its impacts or established guidelines for replacement beef heifers. Current recommendations adopted by FASS (2020) and for feedlot cattle (Henry et al. 2007) are largely derived from dated sources (MWPS 1987). Accordingly, Tucker et al. (2015) highlighted the need for research to define appropriate stocking densities for intensive beef systems, as has been accomplished in other livestock species reared in intensive systems (Estevez et al. 2007). Hence, this experiment was conducted to provide initial data on the effects of different stocking densities on welfare and puberty responses of beef heifers reared in drylots.

### Heifer physical activity and growth responses

Heifer age on d 0 and initial shrunk BW are reported in Table 2 and did not differ (*P *≥ 0.81) among treatments. Table 2 also summarizes physical activity variables recorded using the CowManager system, and treatment effects were detected for time spent eating, non-active, active, and highly active (*P *≤ 0.05). No treatment effects were noted (*P *= 0.76) for time spent ruminating. More specifically, CON heifers spent more time eating (*P < *0.01) and less time being non-active (*P < *0.01), active (*P < *0.01), and highly active (*P ≤ *0.05) compared with heifers reared in drylots. No differences were detected (*P *≥ 0.14) among HDENS, MDENS, and LDENS heifers for time spent eating, non-active, active, and highly active.

**Table 2:** Growth responses and physical activity in replacement beef heifers reared in pastures (1,380 m^2^/heifer; CON, *n* = 6), or in drylots with low stocking density (LDENS; 42 m^2^/heifer, *n* = 6), medium stocking density (MDENS; 28 m^2^/heifer, *n* = 6), or high stocking density (HDENS; 14 m^2^/heifer, *n* = 6) for 170 d.^1^

Item	CON	LDENS	MDENS	HDENS	SEM	*P*
**Age (d 0), d**	271	273	269	271	3	0.81
**Growth responses^3^**						
**Initial shrunk BW (d** −**3), kg**	232	232	232	232	3	0.99
**Final shrunk BW (d 171), kg**	345	343	349	347	8	0.95
**Average daily gain, kg/d**	0.641	0.628	0.663	0.655	0.038	0.92
**Physical activity**						
**Time spent, %^2^**						
**Active**	8.79^b^	12.0^a^	11.2^a^	10.9^a^	0.5	<0.01
**Eating**	21.0^a^	14.3^b^	14.2^b^	14.1^b^	0.6	<0.01
**Highly active**	18.0^b^	19.6^a^	19.4^a^	20.0^a^	0.5	0.05
**Not active**	25.2^b^	27.3^a^	28.8^a^	27.6^a^	0.5	<0.01
**Ruminating**	27.0	26.7	27.3	27.5	0.7	0.76

1 Within rows, values with different superscripts differ (*P *≤ 0.05).

2 Activity parameters were evaluated as in Wolfger et al. (2015) according to the CowManager system (Select Sires, Plain City, OH). Results are reported according to % of the day performing each activity.

3 Calculated using initial (d −3) and final (d 171) shrunk BW, which was recorded after 16 h of feed and water withdrawal.

Although heifers from all treatments received the same limit-fed diet throughout the experiment and pastures were regularly mowed to ensure negligible forage availability, slight forage regrowth likely elicited grazing-related head and neck movements in CON heifers that were classified as time spent eating (Wolfger et al. 2015; Pereira et al. 2018). Accordingly, the increased eating time in CON heifers occurred at the expense of time spent nonactive, active, and highly active, rather than reflecting heightened locomotor activity. Harvey et al. (2024) similarly reported greater time spent eating and reduced time spent non-active in pasture-reared heifers compared with drylot cohorts, although pasture heifers in that study exhibited greater time spent active. Perhaps the larger 2-ha pasture used in Harvey et al. (2024) provided greater opportunity for sustained locomotion compared with the smaller 1.38-h pastures used herein. Hence, the CowManager system effectively distinguished behavioral differences between heifers reared on pasture or in drylots but did not detect behavioral differences among drylot stocking densities. This latter outcome does not indicate that physical activity was unaffected by drylot stocking density, as the CowManager system was also unable to detect differences in physical activity between drylot heifers exposed or not to an exercise regimen 3 times/week (Harvey et al. 2024).

No treatment effects were detected (*P *≥ 0.92) for heifer ADG and final shrunk BW (Table 2). The growth rate of each heifer, modeled using linear regression of full BW and sampling days, also did not differ (*P *= 0.80) among treatments (0.767, 0.742, 0.778, and 0.759 kg/d for CON, LDENS, MDENS, and HDENS, respectively; SEM = 0.020). These outcomes are consistent with previous research (Schubach et al. 2017; Harvey et al. 2024) and the similar nutritional management imposed across treatments. Others have reported greater ADG when heifers are managed in drylots compared with pastures and associated these responses with differences in physical activity (Petersen et al. 2014; Perry et al. 2015). However, drylot heifers in those studies were offered a TMR, whereas pasture-reared cohorts did not receive supplementation beyond grazed forage. Schubach et al. (2017) also proposed that altered physical activity could impact net energy requirements for maintenance but reported similar ADG in drylot heifers compared with cohorts reared on pastures. Hence, these studies support that differences in physical activity among pasture and drylot treatments herein were not sufficient to impact heifer growth.

### Physiological parameters

#### Hair cortisol

**Figure 1:**
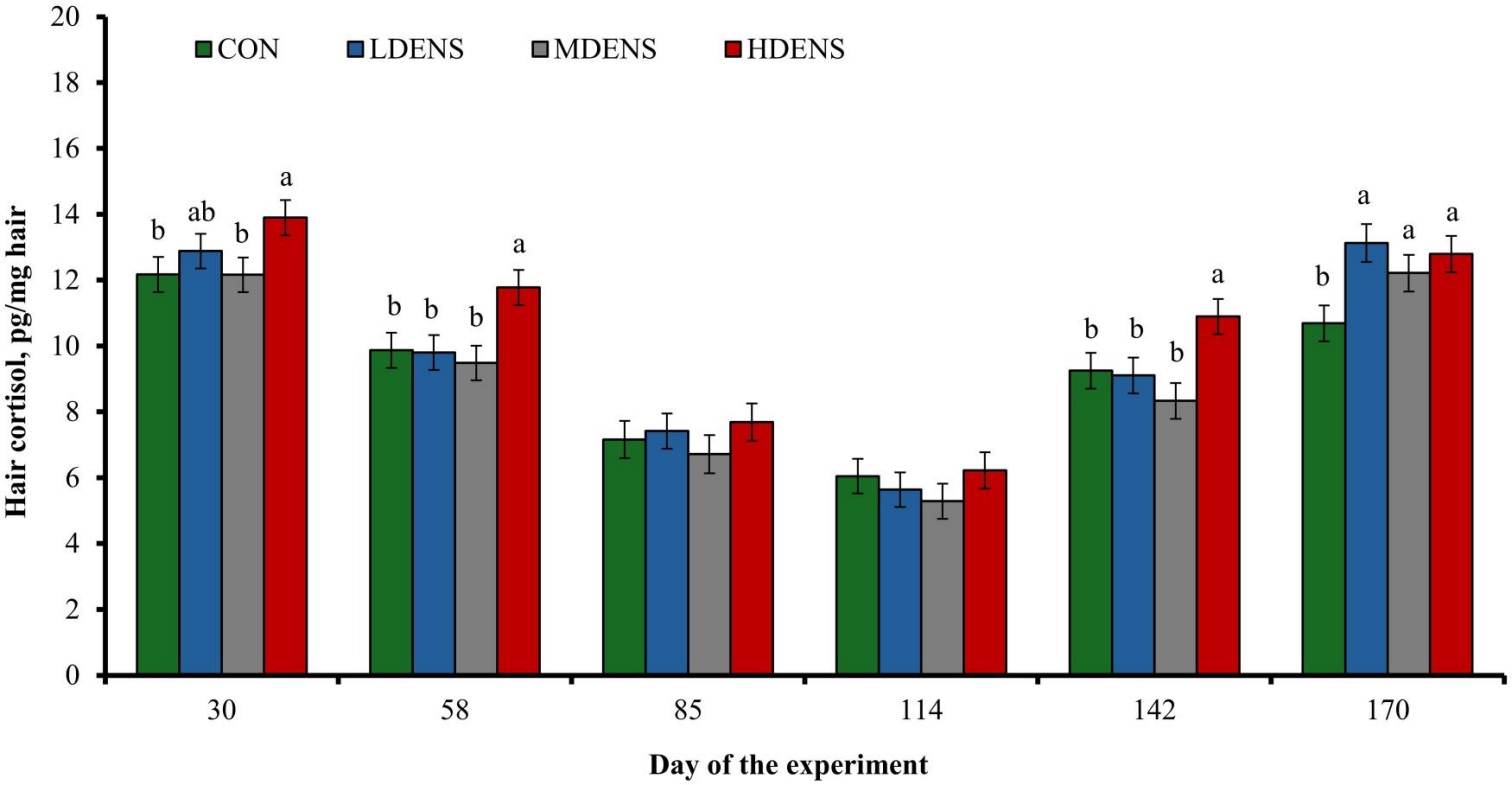
Hair cortisol concentrations in replacement beef heifers reared in pastures (1,380 m^2^/heifer; CON, *n* = 6), or in drylots with low stocking density (LDENS; 42 m^2^/heifer, *n* = 6), medium stocking density (MDENS; 28 m^2^/heifer, *n* = 6), or high stocking density (HDENS; 14 m^2^/heifer, *n* = 6) for 170 d in Exp. 1. Hair samples were collected on days 0, 30, 58, 85, 114, 142, and 170 as in Schubach et al. (2017). Results from day 0 were used as independent covariate. A tendency for a treatment × day interaction was detected (*P *= 0.08). Within days, means with different superscripts differ (*P *≤ 0.05).

A tendency for a treatment × day interaction was detected (*P *= 0.08) for hair cortisol concentrations (Figure 1), which were greater (*P *≤ 0.02) for HDENS compared with CON and MDENS heifers on d 30, greater (*P *≤ 0.04) for HDENS heifers compared with all other treatments on d 58 and 142, and less (*P *≤ 0.05) for CON heifers compared with all drylot treatments on d 170. No other treatment differences were noted (*P *≥ 0.17) within days.

Cortisol measured in tail-switch hair has been used as biomarker of chronic stress in cattle (Moya et al. 2013), given that cortisol accumulates gradually in hair as it grows and its concentration represents long-term adrenocortical activity (Burnett et al. 2014; Cooke et al. 2017). Based on this rationale, HDENS heifers experienced greater chronic stress during most of the study, whereas differences among drylot treatments were no longer detected by the end of the experimental period. These findings suggest that high stocking density (14 m^2^/heifer) elicited an earlier chronic stress response compared with intermediate drylot stocking densities, whereas prolonged exposure to drylot housing promoted chronic stress regardless of stocking density level. The LDENS and MDENS treatments may have required more time to be perceived as a stressor compared with HDENS (Schubach et al. 2017; Schubach et al. 2020; Harvey et al. 2024). In contrast, heifers on pasture experienced less chronic stress early in the experiment compared with HDENS and compared to all drylot treatments during the latter stages of the experiment. Hence, reducing the stocking density from 14 m^2^/heifer to 28 or 42 m^2^/heifer deferred but did not prevent the onset of chronic stress in drylot heifers.

#### Whole blood cells, mRNA expression

**Figure 2:**
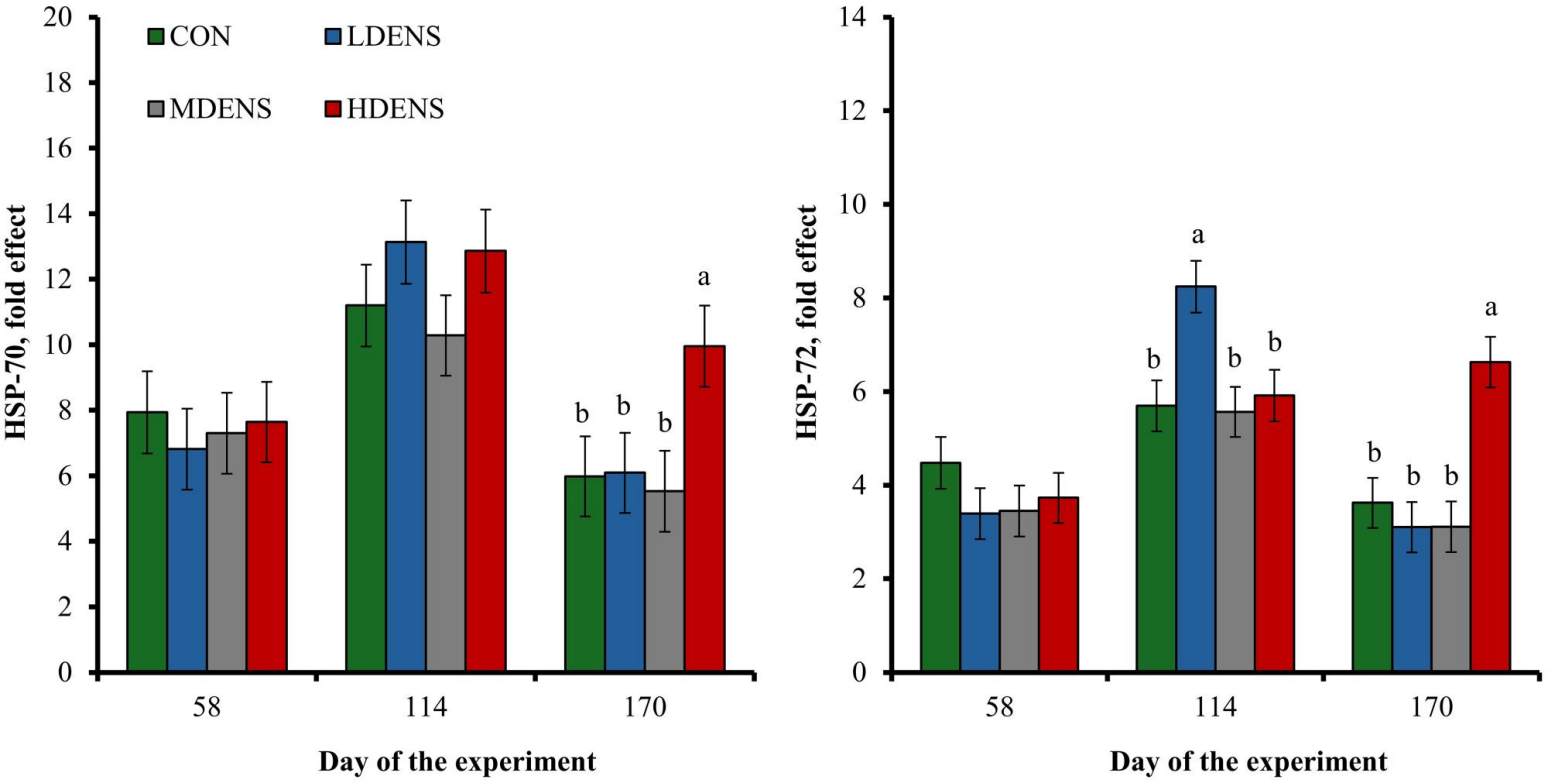
mRNA expression of *heat shock protein 70* (HSP70) and *heat shock protein 72* (HSP72) in whole blood of replacement beef heifers reared in pastures (1,380 m^2^/heifer; CON, *n* = 6), or in drylots with low stocking density (LDENS; 42 m^2^/heifer, *n* = 6), medium stocking density (MDENS; 28 m^2^/heifer, *n* = 6), or high stocking density (HDENS; 14 m^2^/heifer, *n* = 6) for 170 d in Exp. 1. Samples were collected on days 0, 58, 114, and 170 of the experiment, processed as in Pohler et al. (2017), evaluated for mRNA expression according to Rodrigues et al. (2015), and reported as in Ocón-Grove et al. (2008). Data were analyzed using results from day 0 as independent covariate in each respective analysis. A treatment × day interaction was detected (*P *≤ 0.04) for both variables. Within days, means with different superscripts differ (*P *≤ 0.05).

A treatment × day interaction was detected (*P *= 0.04) for blood mRNA expression of *heat shock protein* (HSP)-70 (Figure 2), which was greater (*P *≤ 0.02) for HDENS heifers compared with all other treatments on d 170. A treatment × day interaction was also detected (*P *< 0.01) for mRNA expression of HSP-72 (Figure 2), which was greater (*P *< 0.01) for LDENS heifers compared with all other treatments on d 114 and greater (*P *< 0.01) in HDENS heifers compared with all other treatments on d 170. No other treatment differences were detected (*P *≥ 0.19) for mRNA expression of HSP-70 and HSP-72 during the experiment.

The mRNA expression of HSP in whole blood can be used as a diagnostic marker of stress given that HSP synthesis increases rapidly when cells are exposed to a variety of stressors (Welch 1992). Treatment differences detected for HSP-70 and HSP-72 mRNA expression corroborate the stress consequences of rearing heifers in drylots with high stocking density (14 m^2^/heifer) toward the end of the experimental period. These findings are consistent with Harvey et al. (2024), who reported greater HSP-70 and HSP-72 mRNA expression in drylot heifers compared with pasture heifers mainly after day 168 of the study. The LDENS and MDENS heifers herein exhibited HSP-70 and HSP-72 mRNA expression similar to CON heifers on d 170, indicating that reducing stocking density may have mitigated cellular stress responses associated with prolonged drylot housing. The isolated increase in HSP-72 mRNA expression in LDENS heifers on day 114 was transient and not accompanied by corresponding changes in HSP-70 mRNA expression or hair cortisol concentrations, denoting that this response was likely unrelated to stocking density. Collectively, reducing stocking density attenuated the cellular stress response to drylot housing, whereas high stocking density elicited a more pronounced stress response late in the experimental period.

### Temperament responses

Heifer temperament responses are reported in Table 3, whereas no treatment effects were detected (*P *≥ 0.16) for chute score, exit velocity, and temperament score. No treatment × day interactions were detected for these temperament variables (*P *≥ 0.42). Facilitating increased human-animal interaction, such as rearing cattle in drylot systems or frequent handling, is known to impact cattle temperament (Cooke et al. 2009; Cooke et al. 2012). In Harvey et al. (2024), heifers reared on pasture had less exit velocity and temperament score compared with drylot heifers at the end of the 224-d study, which was interpreted as a behavioral consequence of accumulated chronic stress and heightened anxiety in drylot heifers. Perhaps the 170-d duration of the present experiment was insufficient to elicit detectable differences in temperament among treatments, particularly between CON and HDENS heifers. Moreover, temperament score captures the animal’s response to a specific situation or point in time and alone does not provide an indication of affective or welfare state (Lee et al. 2018; Hubbard et al. 2019). Hence, rearing heifers on pasture or in drylots with differing stocking densities did not impact temperament responses during the 170-d experimental period. Treatment effects on chronic stress indicators suggest that physiological measures were more sensitive than chute-based behavioral assessments for detecting stress-related consequences of stocking density under the conditions of this experiment.

**Table 3.** Temperament and puberty responses in replacement beef heifers reared in pastures (1,380 m^2^/heifer; CON, *n* = 6), or in drylots with low stocking density (LDENS; 42 m^2^/heifer, *n* = 6), medium stocking density (MDENS; 28 m^2^/heifer, *n* = 6), or high stocking density (HDENS; 14 m^2^/heifer, *n* = 6) for 170 d.^1^

Item	CON	LDENS	MDENS	HDENS	SEM	*P*
**Temperament variables^2^**						
**Chute score**	1.35	1.47	1.46	1.34	0.07	0.50
**Exit velocity (m/s)**	2.61	2.56	2.53	2.44	0.10	0.66
**Temperament score**	2.24	2.29	2.23	2.01	0.09	0.16
**Final puberty attainment, %^3^**	73.3	66.7	61.7	61.0	4.8	0.24
**Age at puberty, d**	370^b^	391^a^	392^a^	399^a^	7	0.04
**Body weight at puberty, kg**	311	325	331	331	8	0.29

1 Within rows, values with different superscripts differ (*P *≤ 0.05).

2 According to the techniques described by Cooke (2014). Evaluated on d 0, 85, and 170 of the experiment. No treatment × day interactions were detected for temperament variables (*P *≥ 0.42).

3 Evaluated according to plasma progesterone concentrations in samples collected every 7 d from d 0 to 170 (Cooke and Arthington 2009).

### Puberty attainment

**Figure 3.**
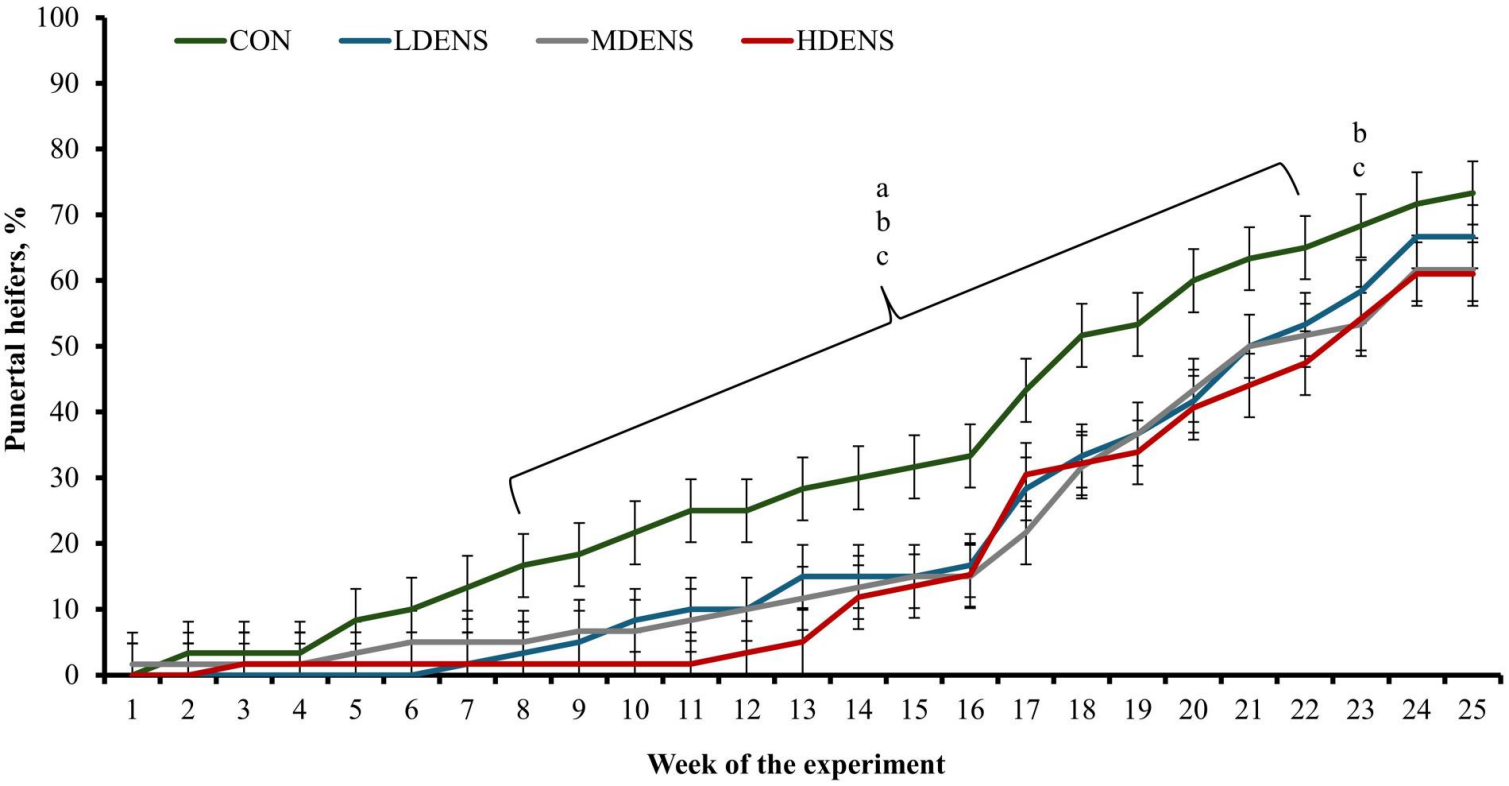
Puberty attainment in replacement beef heifers reared in pastures (1,380 m^2^/heifer; CON, *n* = 6), or in drylots with low stocking density (LDENS; 42 m^2^/heifer, *n* = 6), medium stocking density (MDENS; 28 m^2^/heifer, *n* = 6), or high stocking density (HDENS; 14 m^2^/heifer, *n* = 6) for 170 d in Exp. 1. Puberty was evaluated according to plasma progesterone concentrations in samples collected weekly from days 0 to 170. Heifers were considered pubertal once plasma progesterone concentrations were ≥ 1.5 ng/mL followed by a cyclic pattern of plasma progesterone and < and ≥ 1.5 ng/mL suggestive of normal estrous cycles (Cooke and Arthington 2009). A treatment × day interaction was detected (*P *< 0.01). Within weeks, superscripts indicate treatment differences (*P *≤ 0.05) between: a = CON vs LDENS, b = CON vs. MDENS, c = CON vs. HDENS.

A treatment × day interaction was detected (*P *< 0.01) for puberty attainment (Figure 3). A greater (*P *≤ 0.05) proportion of CON heifers were pubertal by wk 22 of the experiment compared with all other treatments and compared with MDENS and HDENS heifers on wk 23. No other treatment differences were noted within weeks for puberty attainment (*P *≥ 0.17). Final puberty attainment and BW at puberty did not differ (*P *≥ 0.24) among treatments (Table 3); however, age at puberty was less (*P *= 0.01) in CON heifers compared with all other treatments and did not differ (*P *≥ 0.43) among HDENS, MDENS, and LDENS heifers.

Age at puberty in cattle is highly regulated by BW and body composition (Schillo et al. 1992). Rearing heifers in drylots with high stocking density delayed puberty attainment compared with heifers reared on pasture herein and in our previous studies (Schubach et al. 2017; Harvey et al. 2024), despite similar BW gain among treatments. Delayed puberty in HDENS heifers can be attributed to the greater chronic stress experienced by these heifers, as denoted by treatment differences observed for hair cortisol concentration and blood mRNA expression of HSP-70 and HSP-72. Synthesis of gonadotropins necessary for follicular development and ovulation is impaired by chronic and cellular stress responses, particularly increased adrenocortical activity that results in cortisol secretion (Dobson and Smith 2000). Delayed puberty, however, was not alleviated by decreasing stocking density in drylot heifers. This occurred despite MDENS and LDENS exhibiting a deferred onset of chronic stress based on hair cortisol concentrations and a lessened cellular stress response according to blood mRNA expression of HSP-70 and HSP-72 on day 170. Therefore, decreasing stocking density within drylot systems was sufficient to attenuate physiological stress responses but was not adequate to normalize reproductive development relative to heifers reared on pasture.

## Conclusions

Replacement beef heifers reared in drylots with a high stocking density (14 m^2^/heifer) experienced heightened stress-related physiological responses and delayed puberty attainment compared to heifers reared on 1.38-ha pastures with low stocking density. These outcomes were independent of heifer nutritional status and growth rate and were associated with chronic stress caused by high stocking density. Decreasing the stocking density in drylot heifers to 28 m^2^/heifer or 42 m^2^/heifer attenuated chronic and cellular stress responses, without consistent differences between these stocking density levels, but did not translate into hastened puberty attainment. Results from this experiment are novel and demonstrate the need to further explore stocking density levels and other management strategies that improve welfare and reproductive development of beef heifers reared in intensive drylot systems. Future research should evaluate forage regrowth and grazing contribution (e.g. paired clipped and unclipped plots) in CON pastures, drylot pen surface conditions, and subsequent reproductive performance to better inform stocking density recommendations and potential updates to FASS (2020) for replacement heifers.

## Abbreviations

ADG: average daily gain
BW: body weight
CT: threshold cycle
HSP: heat shock protein
TMR: total mixed ration
PCR: polymerase chain reaction
